# Exploring Acculturation Experiences of Korean Immigrant Older Adults Through Expressive Writing

**DOI:** 10.1093/geroni/igaa057.1631

**Published:** 2020-12-16

**Authors:** Stephanie Rhee

**Affiliations:** University of Wisconsin-Green Bay, Green Bay, Wisconsin, United States

## Abstract

Korean immigrant older adults residing in areas without well-established Korean ethnic enclaves experience acculturative stress and depressive symptoms due to their lingual and cultural barriers. Expressive writing can be used as a culturally sensitive intervention to help those immigrants disclose their deepest thoughts and feelings related to their immigration and acculturation experiences. This study gathered qualitative data from the author’s experimental study using expressive writing for first-generation Korean immigrant adults 60 to 88 years of age residing in Midwestern cities. Participants were instructed to write for 15-20 minutes per day in three consecutive days at their convenience in a comfortable and private setting and asked to return their writings by mail. A total of 22 participants returned their writings: 14 wrote about their past and current stressful experiences related to their immigration and acculturation, while eight wrote about their daily lives. Eight themes emerged from thematic coding processes guided by the grounded theory approach: (1) survival, resilience, hardiness, tenacity, pride; (2) lingual barriers; (3) religious faith; (4) gender difference in roles and values; (5) racial discrimination; (6) traditional strategies of acculturation; (7) family and intergenerational gap; and (8) aging. The themes illustrate the participants’ lifelong efforts to shape their unique voices through heart-wrenching struggles, haunting wounds, and ethnic pride and resilience. The study findings suggest that culturally relevant programs and services are needed to facilitate social relationships and reduce lingual and cultural barriers for Korean immigrant older adults residing in non-ethnic enclaves.

